# Impact of the COVID-19 pandemic lockdown on the utilization of acute surgical care in the State of Salzburg, Austria: retrospective, multicenter analysis

**DOI:** 10.1007/s10353-021-00692-1

**Published:** 2021-03-04

**Authors:** Jaroslav Presl, Martin Varga, Christof Mittermair, Stefan Mitterwallner, Michael Weitzendorfer, Ana Gabersek, Kurosch Borhanian, Andreas Heuberger, Helmut Weiss, Klaus Emmanuel, Burkhard von Rahden, Oliver Owen Koch

**Affiliations:** 1 Department of Surgery, Paracelsus Medical Private University (PMU) Salzburg, University Hospital Salzburg (SALK), Müllner Hauptstr. 48, 5020 Salzburg, Austria; 2 Department of Surgery, Saint John of God Hospital, Teaching Hospital of the Paracelsus Medical Private University (PMU) Salzburg, Kajetanerpl. 1, 5010 Salzburg, Austria; 3 Department of Surgery, Hospital Oberndorf, Teaching Hospital of the Paracelsus Medical Private University (PMU) Salzburg, Paracelsusstrasse 37, 5110 Oberndorf, Austria

**Keywords:** SARS-CoV‑2, Pandemic, Emergency, Appendectomy, Colorectal surgery

## Abstract

**Background:**

Some medical disciplines have reported a strong decrease of emergencies during the coronavirus disease 2019 (COVID-19) pandemic; however, the effect of the lockdown on general surgery emergencies remains unclear.

**Methods:**

This study is a retrospective, multicenter analysis of general surgery emergency operations performed during the period from 1 March to 15th 2020 lockdown and in the same time period of 2019 in three medical centers providing emergency surgical care to the area Salzburg-North, Austria.

**Results:**

In total 165 emergency surgeries were performed in the study period of 2020 compared to 287 in 2019. This is a significant decrease of 122 (42.5%) emergency surgeries during the COVID-19 lockdown (*p* = 0.005). The length of hospital stay was reduced to 3 days in 2020 compared to 4 in 2019. Appendectomy remained the most performed emergency surgery for both periods; however the number of surgeries was reduced to less than a half, with 72 cases in 2019 and 33 cases in 2020 (*p* = 0.118). Emergency colon surgery observed the strongest decrease of 75% from 17 cases in 2019 to 4 in 2020. In addition, the emergency abdominal wall hernia, cholecystectomies for acute cholecystitis, small surgeries and proctological emergencies recorded drops of 70%, 39%, 33% and 47% respectively. The strongest reduction in frequencies of emergency surgeries was reported from the designated COVID center in the examined region.

**Conclusions:**

Emergency general surgery is an essential service that continues to run under all circumstances. Our data show that COVID-19-related restrictions have resulted in a significant decrease in the utilization of acute surgical care.

## Main novel aspects

This study demonstrates the negative impact of the COVID-19 pandemic on the utilization of the acute surgical care and its potential effects.

## Introduction

Emergency general surgery encompasses the care of critically ill surgical patients. Rapid diagnosis and accurate therapy of an abdominal emergency can prevent a high lethality of potential intra-abdominal infection, which can reach up to 40% in case of abdominal sepsis [1–4].

When the first cases of a new viral pneumonia—today known as coronavirus disease 2019 (COVID-19) caused by severe acute respiratory syndrome coronavirus 2 (SARS-CoV-2)—were reported from Wuhan/China in December 2019, nobody knew that in March 2020 the COVID-19 would become a pandemic [5]. The high mortality of the disease, which initially reached 7.2% in Italy [6], forced authorities, due to a lack of causal therapy or vaccination, to declare a lockdown in order to slow the spread. The first COVID-19-positive case in the State of Salzburg, Austria was documented on 29 February 2020. Because of the growing caseload of COVID-19-positive patients, the government took the first steps to restrict public life on 16 March 2020. These measures were gradually tightened until 14 April 2020 when parts of the restrictions were withdrawn. In order to avoid the spread in the public hospitals and to spare health care capacities, all elective interventions (including surgeries) were postponed and the population was encouraged to avoid the hospitals except for emergencies. Simultaneously with the increasing numbers of infected patients and growing fear of possible overwhelming numbers of COVID-19 patients, another effect of the lockdown was reported from abroad. In the USA, the nine high-volume catheterization laboratories reported a decrease activation for ST-elevation myocardial infarction (STEMI) of 38% [7]. The similar trend could be observed by neurologists from China and USA, who reported a dramatic decline of acute strokes by 30–50% [8, 9].

Currently, there is no knowledge about the effect of the lockdown on the presentation of the general surgery emergencies, which could be crucial to prevent development of severe adverse effects. The aim of this work is to investigate whether there have been any changes in surgical emergency operations in the northern area of the State of Salzburg, Austria (supply region 51) during the national lockdown.

## Methods

We have conducted a retrospective analysis of all consecutive general surgery emergency surgeries performed in the entire emergency region 51 in the period from 1 March to 15 April 2020 (6 weeks). Region 51 is a defined northern area of the State of Salzburg, Austria with 364,797 inhabitants, which is 66% of the population of the whole state of Salzburg. There are only three public general surgery centers providing emergency surgical care in this region:

- Center 1: The Department of Surgery of the Paracelsus Medical Private University (PMU) Salzburg. This center also includes the data of its external Surgical Division located in the city of Hallein, Austria (PMU+Hallein).
- Center 2: Saint John of God Hospital, Teaching Hospital of the Paracelsus Medical Private University (PMU) Salzburg (BHB).
- Center 3: Department of Surgery, Hospital Oberndorf, Teaching Hospital of the Paracelsus Medical Private University (PMU) Salzburg, Oberndorf, Austria (Oberndorf).

Demographic data (age, sex), the length of hospital stay (LOS), the type of acute surgery, the indication for surgery and the mortality rates were retrospectively gathered from the clinical databases and analyzed. For comparison, the same parameters from the three centers in the time period 1 March to 15 April of the year 2019 were used. The surgeries were divided into 13 main categories, defined upon the most frequent clinical diagnosis, according to which the statistic comparison followed (appendectomy, cholecystectomy, bowel ischemia, small bowel perforation, acute abdominal wall hernia, bowel obstruction, small acute surgeries = small one day surgeries, colon emergencies, proctological emergencies, revision operations after any index operation, emergency thorax surgery, abdominal trauma surgeries, stomach bleeding or perforation surgeries). Only Center 1 was dedicated as a regional COVID center (“hot” hospital) so it covered the care of COVID and non-COVID patients. This took place in a dedicated COVID clinic located on the campus of the University Hospital in Salzburg [10]. Centers 2 and 3 remained COVID free during the lockdown. All patients included in the study were considered COVID-19 negative.

## Statistical analysis

Statistical analysis was performed using SPSS Statistical Analysis Software (SPSS Inc., Chicago, IL, USA). All data were tested for normal distribution by the Kolmogorov–Smirnov test. Data comparisons were done using paired *t*-test or Wilcoxon signed rank test on a per subject basis. Population homogeneity was conducted using independent *t*-test or Mann–Whitney U test. If normally distributed, measurements were additionally presented as means and standard deviation (SD); *p* < 0.05 was regarded as statistically significant.

## Results

**Fig. 1:**
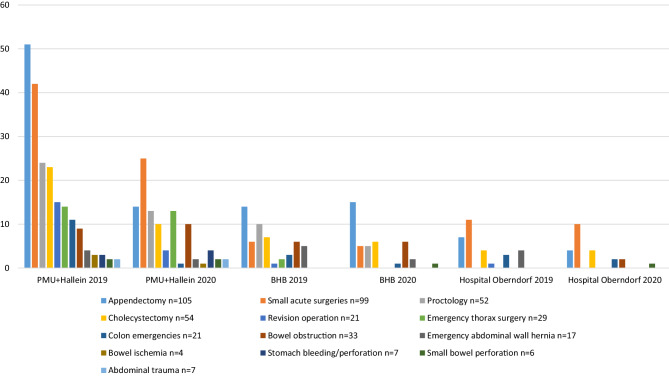
Emergency surgeries in 2019 vs. 2020. *PMU+Hallein *The Department of Surgery of the Paracelsus Medical Private University Salzburg, this center also includes the data of its external Surgical Division located in the city of Hallein, *BHB* Saint John of God Hospital Salzburg

In region 51, 165 emergency surgeries were performed in the study period of 2020 compared to 287 emergency surgeries in 2019. This is a significant decrease of 122 (42.5%) emergency surgeries during the COVID-19 lockdown (*p* = 0.005; Table 1). In total, 248 (54.9%) male and 204 (45.1%) female patients underwent an emergency operation in 2019 and 2020. The distribution according to gender remained equal in 2019 (144 male vs. 143 female). During the COVID-19 lockdown in 2020, 104 male patients (63%) underwent surgery compared to 61 female patients (37%). The mean age of the patients who underwent surgery in 2019 vs 2020 was 52 years (±20.3) and 52 years (±20.1), respectively, and was not significantly different (*p* = 0.991). Comparing the length of hospital stay (LOS) between the year 2019 and 2020 in each individual center, we have not found any significant difference; however the median LOS in 2020 was reduced from 4 to 3 days in comparison to the year before (Table 2). Appendectomy remained the most frequently performed emergency surgery for both time periods, but the number of operations decreased from 72 cases to less than a half with 33 cases in 2020 (*p* = 0.118). Considering the ratio of appendectomy vs all emergency surgeries, it represented 25% in 2019 and 20% in 2020. A statistically significant decrease from 51 appendectomies in 2019 to 14 in 2020 (*p* = 0.047) was reported only in Center 1 (the “hot” hospital). The second most frequent surgery were small surgeries (including surgery for small skin inflammation—excluding proctology), showing a decrease of 20% (*p* = 0.156; 59 procedures in 2019 and 40 in 2020). Emergency colon surgery observed the strongest decrease of 75% (17 cases in 2019 to 4 in 2020). Again, Center 1 reported a decrease from 11 cases in 2019 to only 1 case in 2020 (Table 1). The number of cholecystectomies in case of acute cholecystitis dropped from 33 operations to 20 during the lockdown (*p* = 0.262). Center 1 reported a decrease of more than 62%. In general, Center 1 recorded significant decreases in 6 of the main categories of the surgeries (Table 1; Fig. 1).

**Table 1:** Distribution of emergency surgeries in the three centers according to the 13 main categories

		Period 01 March–15 April 2019	Period 01 March–15 April 2020	*p*-value
Emergency operations	Total	287	165	*p* = 0.005
*Appendectomy*	*n* *=* *105*	*72*	*33*	*p* = 0.118
PMU+Hallein		51	14	*p* = 0.047
BHB		14	15	*p* = 0.970
Hospital Oberndorf		7	4	*p* = 0.438
*Cholecystectomy*	*n* *=* *54*	*33*	*20*	*p* *=* *0.262*
PMU+Hallein		23	10	–
BHB		7	6	–
Hospital Oberndorf		4	4	–
*Bowel ischemia*	*n* *=* *4*	–	–	–
PMU+Hallein		3	1	–
*Small bowel perforation*	*n* *=* *6*	*2*	*4*	–
PMU+Hallein		2	2	–
BHB		0	1	–
Hospital Oberndorf		0	1	–
*Emergency abdominal wall hernia*	*n* *=* *17*	*13*	*4*	*p* = 0.425
PMU+Hallein		4	2	–
BHB		5	2	–
Hospital Oberndorf		4	0	–
*Bowel obstruction*	*n* *=* *33*	*15*	*18*	*p* = 0.324
PMU+Hallein		9	10	–
BHB		6	6	–
Hospital Oberndorf		0	2	–
*Small acute surgeries*	*n* *=* *99*	*59*	*40*	*p* *=* *0.156*
PMU+Hallein		42	25	*p* = 0.225
BHB		6	5	–
Hospital Oberndorf		11	10	–
*Colon emergencies*	*n* *=* *21*	*17*	*4*	*p* = 0.421
PMU+Hallein		11	1	–
BHB		3	1	–
Hospital Oberndorf		3	2	–
*Proctology*	*n* *=* *52*	*34*	*18*	*p* = 0.271
PMU+Hallein		24	13	–
BHB		10	5	–
Hospital Oberndorf		0	0	–
*Revision operation*	*n* *=* *21*	*17*	*4*	*p* *=* *0.421*
PMU+Hallein		15	4	–
BHB		1	0	–
Hospital Oberndorf		1	0	–
*Emergency thorax surgery*	*n* *=* *29*	*16*	*13*	*p* *=* *0.342*
PMU+Hallein		14	13	–
BHB		2	0	–
Hospital Oberndorf		0	0	–
*Abdominal trauma*	*n* *=* *4*	–	–	–
PMU+Hallein		2	2	–
*Stomach bleeding/perforation*	*n* *=* *7*	*3*	*4*	–
PMU+Hallein		3	4	–
BHB		0	0	–
Hospital Oberndorf		0	0	–

Italic numbers denote the total number per the category and year

*PMU+Hallein *The Department of Surgery of the Paracelsus Medical Private University Salzburg, this center also includes the data of its external Surgical Division located in the city of Hallein, BHB Saint John of God Hospital Salzburg

**Table 2:** Demographic data, length of stay and mortality

	Period 01 March–15 April 2019	Period 01 March–15 April 2020	Total	*p*-value
*Age*	*Median*	*Median*	*–*	*–*
Median	52 (± 20.3)	52 (± 20.1)	–	*p* = 0.991
PMU+Hallein	51 (± 20.5)	54 (± 19.8)	–	*p* = 0.867
BHB	54 (± 20.9)	51 (± 19.2)	–	*p* = 0.859
Hospital Oberndorf	51.5 (± 21.8)	43 (± 19.0)	–	*p* = 0.678
*Gender*	*Male (Female)*	*Male (Female)*	*Male (Female)*	
Total	144 (143) = *287*	104 (61) = *165*	*248 (204)* *=* *452*	*p* *=* *0.005*
PMU+Hallein	104 (99)	71 (30)	175 (129) = *304*	–
BHB	22 (32)	23 (18)	45 (50) = *95*	–
Hospital Oberndorf	18 (12)	10 (13)	28 (25) = *53*	–
*LOS*	*Median*	*Median*	–	–
Median	4 (0–79)	3 (0–49)	–	–
PMU+Hallein	3.5 (± 15.9)	3.4 (± 8.1)	–	*p* = 0.917
BHB	4 (± 5.7)	3 (± 8.6)	–	*p* = 0.873
Hospital Oberndorf	3 (± 15.3)	5 (± 6.)	–	*p* = 0.724
*Mortality*	–	–	–	–
PMU+Hallein	12	1	–	–
BHB	2	0	–	–
Hospital Oberndorf	0	0	–	–

Italic numbers denote the total number per category and year

LOS length of stay, *PMU+Hallein *The Department of Surgery of the Paracelsus Medical Private University Salzburg, this center also includes the data of its external Surgical Division located in the city of Hallein, BHB Saint John of God Hospital Salzburg

## Discussion

The COVID-19 pandemic lockdown caused a significant reduction of the general surgery emergency operations of 42.5% (*p* = 0.005) in the entire region 51. This noteworthy reduction of emergencies was also reported from other medical specialties around the globe. Garcia et al. reported about decreased use of US cardiac catheterization laboratories for STEMI in the COVID-19 era [7]. Medscape Medical News also announced a 30% decreased rates of major stroke in Chicago [8]. Furthermore, the preliminary data to stroke care from Shanghai reported a decrease of thrombectomies by 50% in the first month after the Spring Festival (24–30 January 2020) [9]. Those specialties discussed the reduction of stress and the deceleration of public life as a potential reason for decrease of cardiovascular emergencies [7–9].

Whereas the role of negative stress in functional gastrointestinal disorders is well known, the effect of stress on development of abdominal emergencies has not been described so far [11].

The strong decrease of acute appendectomies and acute cholecystectomies of 54% and 39%, respectively, in 2020 in our cohort is striking. Many different deviations of the therapy concepts, to minimize the risk of COVID-19 cross infection due to the prolonged stay in the hospital, are being reported. Patel et al. recently described an increased use of conservative antibiotic therapy for uncomplicated appendicitis, during the lockdown in the UK [12]. This therapy is reported to be a feasible and safe option, but with an increased risk for appendicitis recurrence [13, 14]. Patel also reported a deviation from the guidelines and an exclusive use of a conservative therapy as a first-line treatment for acute cholecystitis [12, 15].

Based on this knowledge, participating centers in region 51 confirmed no deviation in the surgical treatment of acute appendicitis and cholecystitis.

The official recommendation by authorities to the public was to avoid hospital visits until an emergency occurs. Furthermore, the patients were encouraged to initially contact general practitioners if medical assistance was required. However, the general practitioners have not reported any increase of patient load or change of the therapeutic pathways, including the reporting of all patients suspected of abdominal emergency to a surgical specialist. If the reduction of the case load of emergencies or preferred antibiotic treatment or both are responsible for reduction of acute surgeries in our region remains unknown.

Restrictive politics of the authorities, with the aim of preparing the health care system for a huge number of COVID-19-positive patients, as seen in Italy, had a positive effect on rapid transfer of non-COVID patient therapy to the outpatient setting, leading to a shorter median LOS of 3 days (reduction of 25%) in the examined period of 2020. The same trend was reported by Patel et al. in his cohort with a significant reduction of LOS from 3 to 2 days (*p* = 0.04) [12].

Because there are no other centers in region 51 that cover emergency general surgical care, the most likely reasons for why patients generally avoid medical care of emergency surgeries in region 51 are due to either social distancing or due to the concerns of becoming infected with COVID-19 in the hospital. To underline these facts, we identified a decrease in the frequency of 6 of 13 main categories of emergency surgeries in center 1, which was the only COVID designated center (“hot” hospital) in region 51. Huang et al. reported that this potential perception of personal danger dramatically reduced the willingness of patients to visit an emergency department of the “hot” hospital in 2003 during the last epidemic of SARS from Taipei, Taiwan [16]. The authors reported a reduction of up to 51.6% in daily visits to emergency departments at the peak of the SARS epidemic, which persisted for 3 months after the end of the epidemic. The long-lasting effect of people’s fear of designated SARS centers was reported from the same hospital in Taipei and published in 2008. The long-term results show that the outpatient department of the general surgery department were still treating significantly fewer patients 4 years after the SARS epidemic [17].

Further consequences of the COVID-19 pandemic for acute surgical patients who did not receive appropriate, guideline-conform therapy remains unclear and should be further examined.

## Conclusions

Emergency general surgery is an essential health service that continues to run under all circumstances. Our data show that the COVID-19-related restrictions led to a significant decrease in utilization of acute surgical care. The potentially high mortality of the surgical emergencies must be a warning for public health care providers who should encourage people to continue to use public health care services, especially when suffering from serious medical complaints. New policies are needed to ensure safe access to specialized health services for patients 24 hours a day, 7 days a week, especially in situations like those caused by the COVID-19 pandemic.

## Abbreviations

BHB: Saint John of God Hospital, Teaching Hospital of the Paracelsus Medical Private University (PMU) Salzburg
Elderly: Persons who are 65 years and older
ES: Emergency surgeries
LOS: Length of stay
Oberndorf: Department of Surgery, Hospital Oberndorf, Teaching Hospital of the Paracelsus Medical Private University (PMU) Salzburg, Oberndorf, Austria.
PMU+Hallein: The Department of Surgery of the Paracelsus Medical Private University (PMU) Salzburg, this center also includes the data of its external Surgical Division located in the city of Hallein
Region 51: North part of the State of Salzburg, Austria
STEMI: ST-elevation myocardial infarction
